# *In vitro* osteogenic and odontogenic differentiation of human dental pulp stem cells seeded on carboxymethyl cellulose-hydroxyapatite hybrid hydrogel

**DOI:** 10.3389/fphys.2015.00297

**Published:** 2015-10-27

**Authors:** Gabriella Teti, Viviana Salvatore, Stefano Focaroli, Sandra Durante, Antonio Mazzotti, Manuela Dicarlo, Monica Mattioli-Belmonte, Giovanna Orsini

**Affiliations:** ^1^Department of Biomedical and Neuromotor Sciences, University of BolognaBologna, Italy; ^2^1st Orthopaedic and Traumatologic Clinic, Rizzoli Orthopedic InstituteBologna, Italy; ^3^Department of Clinical and Molecular Sciences, Polytechnic University of MarcheAncona, Italy; ^4^Department of Clinical Sciences and Stomatology, Polytechnic University of MarcheAncona, Italy

**Keywords:** osteogenic differentiation, odontogenic differentiation, dental pulp stem cells, hydrogel, tissue engineering

## Abstract

Stem cells from human dental pulp have been considered as an alternative source of adult stem cells in tissue engineering because of their potential to differentiate into multiple cell lineages. Recently, polysaccharide based hydrogels have become especially attractive as matrices for the repair and regeneration of a wide variety of tissues and organs. The incorporation of inorganic minerals as hydroxyapatite nanoparticles can modulate the performance of the scaffolds with potential applications in tissue engineering. The aim of this study was to verify the osteogenic and odontogenic differentiation of dental pulp stem cells (DPSCs) cultured on a carboxymethyl cellulose—hydroxyapatite hybrid hydrogel. Human DPSCs were seeded on carboxymethyl cellulose—hydroxyapatite hybrid hydrogel and on carboxymethyl cellulose hydrogel for 1, 3, 5, 7, 14, and 21 days. Cell viability assay and ultramorphological analysis were carried out to evaluate biocompatibility and cell adhesion. Real Time PCR was carried out to demonstrate the expression of osteogenic and odontogenic markers. Results showed a good adhesion and viability in cells cultured on carboxymethyl cellulose—hydroxyapatite hybrid hydrogel, while a low adhesion and viability was observed in cells cultured on carboxymethyl cellulose hydrogel. Real Time PCR data demonstrated a temporal up-regulation of osteogenic and odontogenic markers in dental pulp stem cells cultured on carboxymethyl cellulose—hydroxyapatite hybrid hydrogel. In conclusion, our *in vitro* data confirms the ability of DPSCs to differentiate toward osteogenic and odontogenic lineages in presence of a carboxymethyl cellulose—hydroxyapatite hybrid hydrogel. Taken together, our results provide evidence that DPSCs and carboxymethyl cellulose—hydroxyapatite hybrid hydrogel could be considered promising candidates for dental pulp complex and periodontal tissue engineering.

## Introduction

The goal of tissue engineering and regenerative medicine is to improve or restore the functions of diseased tissues and organs. Tissue engineering strategies require main elements such as stem cells, scaffold or matrix, and growth factors (Kabir et al., 2014).

Adult mesenchymal stem cells (MSCs) have the potential to renew themselves for long periods through cell division and, under certain physiological or experimental conditions, they can be induced to become specialized cells (Verma et al., 2014; Potdar and Jethmalani, 2015).

Five types of MSCs have been isolated from dental tissues and demonstrated to have high proliferative and multilineage differentiation properties: dental pulp stem cells (DPSCs; Gronthos et al., 2000; Tirino et al., 2011; Pisciotta et al., 2012, 2015; La Noce et al., 2014), stem cells from human exfoliated deciduous teeth (SHEDs; Miura et al., 2003), periodontal ligament stem cells (PDLSCs; Seo et al., 2004), dental follicle progenitor stem cells (DFPCs; Morsczeck et al., 2005) and stem cells from apical papilla (SCAPs; Sonoyama et al., 2008).

Dental pulp is considered a rich source of DPSCs, suitable for tissue engineering applications (La Noce et al., 2014). It has been shown that DPSCs can be differentiated by modulation with growth factors, transcriptional factors, extracellular matrix proteins, and receptor molecules into different cell types including odontoblasts, osteoblasts, chondrocytes, cardiomyocytes, neuron cells, adipocytes, corneal epithelial cells, melanoma cells, and insulin secreting Beta cells (Pisciotta et al., 2012; La Noce et al., 2014; Paino et al., 2014; Potdar and Jethmalani, 2015).

DPSCs isolated from dental pulp co-express typical MSCs markers such as CD44, CD73, CD90, CD105, CD271, and STRO-1, while negative markers are CD34, CD45, and HLA-DR (La Noce et al., 2014; Verma et al., 2014; Potdar and Jethmalani, 2015). Pisciotta et al. (2015) recently demonstrated the co-existence of two subpopulations of adult stem cells derived from human DPSCs, one of mesodermal origin and a second of neural crest derivation, increasing the potential application of DPSCs in regenerative medicine. However, there is no specific or strict marker characterizing DPSCs, which are considered a heterogeneous population.

Recent attention has been focused on the utilization of DPSCs in tissue engineering (d'Aquino et al., 2009a; Verma et al., 2014). Several scaffolds have been used to promote 3-D tissue formation and studies have demonstrated that DPSCs show good adherence and bone tissue formation on microconcavity surface textures (Graziano et al., 2008; Naddeo et al., 2015). In oro-maxillo-facial bone repair DPSCs have been seeded on collagen sponge scaffolds, producing an optimal biocomplex for regeneration of DSPCs for bone repair (d'Aquino et al., 2009b). A 3-year follow-up demonstrated the synthesis of adult bone tissue with good vascularization (Giuliani et al., 2013), showing the powerful application of DPSCs in periodontal and dental pulp tissue engineering.

In tissues engineering strategies, the choice of an appropriate scaffold is a crucial step (Galler et al., 2011). Hydrogels have been employed as an emerging and promising tool in regenerative medicine but also as an injectable filler material (Varma et al., 2014; Vashist and Ahmad, 2015). A variety of natural and synthetic polymers have been used to fabricate hydrogels. Collagen, hyaluronic acid, chondroitin sulfate, fibrin, fibronectin, alginate, agarose, chitosan, and silk have been the most commonly used natural polymers (Geckil et al., 2010). Cellulose is one of the most ubiquitous and abundant of biopolymers produced in the biosphere. Tissue engineering and regenerative medicine researchers have shown an interest in cellulose because it has a low density and an excellent biodegradability associated with low ecotoxicological risks (Domingues et al., 2014). One of the main drawbacks of cellulose-based hydrogels is the lack of good mechanical proprieties (Pasqui et al., 2012).

Recently, a semisynthetic natural polymer obtained from the carboxymethylation of natural cellulose, named carboxymethyl cellulose (CMC) was developed with hydroxyapatite (HA) crystals to generate a new hybrid hydrogel for bone tissue engineering (Pasqui et al., 2014). Although the chemical and mechanical proprieties were well described (Pasqui et al., 2014), data on the biological performance of CMC-HA hydrogels on stem cells differentiation is lacking.

The aim of this study was to investigate the *in vitro* osteogenic and odontogenic differentiation of DPSCs seeded on a CMC-HA hydrogel. DPSCs were seeded on a CMC-HA hydrogel in presence of osteogenic factors for a period of up to 21 days. Biocompatibility was measured by MTT assay, while electron microscopy analyses were carried out to demonstrate cell adhesion on the scaffold surface.

The markers alkaline phosphatase (ALP), Runt-related transcription factor 2 (RUNX2), type I collagen and osteonectin (SPARC) were chosen for the examination of osteogenic differentiation, while the markers dentin matrix protein I (DMP1), and dentin sialophospho protein (DSPP) were chosen for the examination of odontogenic differentiation. Gene expression was tested by Real Time PCR. Results were compared to DPSCs seeded on CMC-based hydrogels and to DPSCs seeded on 2D system.

## Materials and methods

### Scaffold synthesis and characterization

CMC-based hydrogels without and with hydroxyapatite (HA) were synthesized and characterized as already described in Pasqui et al. (2014). Briefly, the crosslinking agent 1,3-diaminopropane (DAP) was added to the mixture at a molar ratio of 0.5 with respect to the moles of carboxylic acid of the polymers and to the activating agents 1-ethyl-3-[3-(dimethyl-amino)propyl)carbodiimide hydrochloride (EDC) and N-hydroxysuccinimide (NHS) moles. The molar ratio of EDC and NHS mole is 1 to 1. The pH of the mixture was adjusted to 4.75 by the addition of HCl 1M solution and the reaction was allowed to go on under stirring overnight at room temperature. Once formed, the hydrogel was purified by dipping it in water for 4–5 days until no traces of free EDC or NHS were revealed by UV spectrophotometry and finally freeze-dried. To load HA inside CMC hydrogel, an exactly weighted amount of freeze-dried hydrogel was dipped in a five solutions containing increasing amounts of HA in a range from 0.5 to 2.5 mg mL. Above this concentration, HA crystals form aggregates which precipitate. The hydrogel was let to swell under moderate stirring for 48 h that is a sufficient time to allow it to reach the maximum swelling degree. Finally, the hydrogel was removed from the solution washed with deionized water until no trace of HA was revealed in the washing solution and freeze-dried again.

### Human dental pulp stem cells (DPSCs) isolation

DPSCs were obtained from healthy permanent premolars extracted during orthodontic treatment, under informed consent. Cells were isolated from dental pulp as described in a previous study (Teti et al., 2013). Briefly, the dental pulp was harvested from the teeth, carefully minced and collected in 60 × 15 mm cell-cultured dishes in Dulbecco's Modified Eagle Medium/F12 (DMEM, Gibco, Life Technologies, Monza, Italy) supplemented with 10% Fetal Bovine Serum (FBS, Gibco, Life Technologies, Monza, Italy) and 1% penicillin/streptomycin and then incubated at 37°C in a humidified atmosphere of 5% CO_2_. At day 7–10 of culture, cells obtained from the minced dental pulp were subsequently trypsinized, resuspended, and plated in T25 tissue flasks in DMEM /F12 medium supplemented with 10% FBS and 1% penicillin/streptomycin. The cells were subcultured once a week using 1% trypsin (Gibco, Life Technologies, Monza, Italy), expanded in new T25 flasks and maintained at 37°C in a humidified atmosphere at 5% CO_2_. Cells from passages 3–5th were utilized for the experiments described.

### FACS analysis

The cells obtained were checked for their staminal profile by FACSCalibur flow cytometry system (Becton Dickinson, CA, USA). In agreement with minimal criteria for the identification of human MSCs (Dominici et al., 2006), 2.5 × 10^5^ cells were removed with Dulbecco Phosphate buffer saline (D-PBS) and were then stained for 45 min with the following antibodies: fluorescein isothiocyanate-(FITC)-labeled mouse anti-human CD90 (StemCell Technologies, Milan, Italy), CD105, CD14, CD19, (Diaclone, France), R-phycoerythrin-(PE)-labeled mouse anti human CD34, CD45 (Diaclone, France), CD73 (Becton Dickinson, CA, USA), and anti HLA-DR (Diaclone, France). The control for FITC- or PE-coupled antibodies was isotypic mouse IgG1. The data were evaluated using CellQuest software (Becton Dickinson, CA, USA).

### Osteogenic differentiation

2 × 10^5^ cells/ 100 μl of medium were seeded on CMC and CMC-HA scaffolds and incubated with the osteogenic medium for 1, 3, 5, 7, 14, and 21 days. The osteogenic medium consisted in alpha MEM medium (Life Technologies, Milan, Italy) supplemented with 10% FBS and 1% penicillin/streptomycin, 250 μmol/L ascorbic acid phosphate, 10 mmol/L beta glycerophosphate, and 10 nmol/L dexamethasone. DPSCs were incubated at 37°C with 5% CO_2_. DPSCs between the 3rd and 5th passages were used throughout the study.

### MTT assay

After each incubation time, the medium was changed to a fresh one containing 0.5 mg/ml of 3-(4,5-dimethylthiazol-2-yl)-2,5-diphenyltetrazolium bromide (MTT) and left for 2 h at 37°C. The formazan produced was dissolved by solvent solution (0.1 N HCl in isopropanol) and the optical density was read at 570 nm by Microplate Reader (Model 680, Biorad Lab Inc., CA, USA).

### High resolution scanning electron microscopy (HR-SEM)

At each experimental point, DPSCs seeded on CMC and CMC-HA scaffolds were washed in 0.15 M sodium cacodylate buffer, fixed with a solution of 2.5% glutaraldehyde in 0.1 M cacodylate buffer (Sigma Aldrich, St. Louis, Missouri, USA) for 2 h at 4°C and subsequently fixed with 1% OsO_4_ in 0.1 M cacodylate buffer (Societa' Italiana Chimici, Roma, Italy) for 1 h at RT (room temperature). After washes in 0.15 M cacodylate buffer, the samples were dehydrated in an ascending alcohol series and CPD (critical point dried 030, Bal-Tec, Leica Microsystems GmbH, Wetzlar, Germany). The samples were then metal coated with a thin layer of carbon/platinum (Bal-Tec, Leica Microsystems GmbH, Wetzlar, Germany) and observed under HR-SEM (JSM 890, Jeol Company, Tokio, Japan) with 10 kV accelerate voltage and 1 × 10^−11^ mA.

### Transmission electron microscopy (TEM)

At each experimental point, DPSCs seeded on CMC and CMC-HA scaffolds were fixed with 2.5% glutaraldehyde in 0.1 M cacodylate buffer for 2 h at 4°C and subsequently post-fixed with 1% OsO_4_ in 0.1 M cacodylate buffer for 1 h at RT. After several washes, the samples were dehydrated in an acetone series (70, 90, 100%) and embedded in LR London white resin (Fluka, Sigma-Aldrich, St. Louis, Missouri, USA). Sections of 100 nm were collected on nickel grids, stained with uranyl acetate and lead citrate, and observed by Philips CM10 (FEI Company, Eindhoven, The Netherlands). Images were recorded by Megaview III digital camera (FEI Company, Eindhoven, The Netherlands). Some sections were stained with toluidine blue staining solution and observed under a light microscopy Nikon Eclipse E800 (Nikon Corporation, Tokyo, Japan).

### RNA extraction and quantitative real time polymerase chain reaction (qRTPCR)

Total RNA was extracted in DPSCs seeded on CMC and CMC-HA scaffolds for 1, 3, 5, 7, 14, and 21 days, by NucleoSpin RNA I kit (Machery-Nagel, Duren, Germany), quantified using a NanoDrop® ND-1000 UV-Vis Spectrophotometer (Thermo Scientific, Wilmington, DE, USA), and cDNA was transcribed with reverse transcriptase SUPIII (Invitrogen, Carlsbad, CA, USA). The expression of mRNA was analyzed by quantitative Real Time PCR using 7500 Real Time PCR (Applied Biosystem, Life Technologies, Monza, Italy). For the analysis, the following TaqMan assays (Applied Biosystems, Life Technologies, Monza, Italy) were used: phosphatase alkaline (ALP Hs01029144_m1), runt-related transcription factor2 (RUNX2 Hs00231692_m1), osteonectin (SPARC Hs00234160_m1), collagen type I alpha 1 (COL1A1 Hs00164004_m1), dentin matrix protein 1 (DMP1 Hs01009390_m1), dentin sialophospho protein (DSPP Hs00171962_m1).

The relative gene expressions were normalized to glyceraldehyde 3-phosphate dehydrogenase (GAPDH Hs99999905_m1), and the data were presented as the fold change using the formula 2^−ΔΔCT^ as recommended by the manufacturer (User Bulletin No.2 P/N 4303859, Applied Biosystems). The gene expression of DPSCs cultured on CMC-HA samples was relative to DPSCs cultured on tissue flasks for 21 days (2D system) in absence of osteogenic medium. Total RNA was extracted as previously described.

Data showed the average of triplicates ± SD and were representative from three independent experiments.

### Statistical analysis

For cell viability data, the statistical differences were assessed by One-way ANOVA (*P* < 0.05) and Dunnet's Multiple Comparison Test (*P* < 0.05). For Real Time data the statistical differences were assessed by Two-way ANOVA (*P* < 0.05) and Bonferroni Multiple Comparison Test (*P* < 0.05).

The statistical analysis was performed with GraphPad Prism 5.0 software (San Diego, CA, USA).

## Results

### Flow cytometry (FACS) analysis of human dental pulp stem cells (DPSCs)

**Figure 1:**
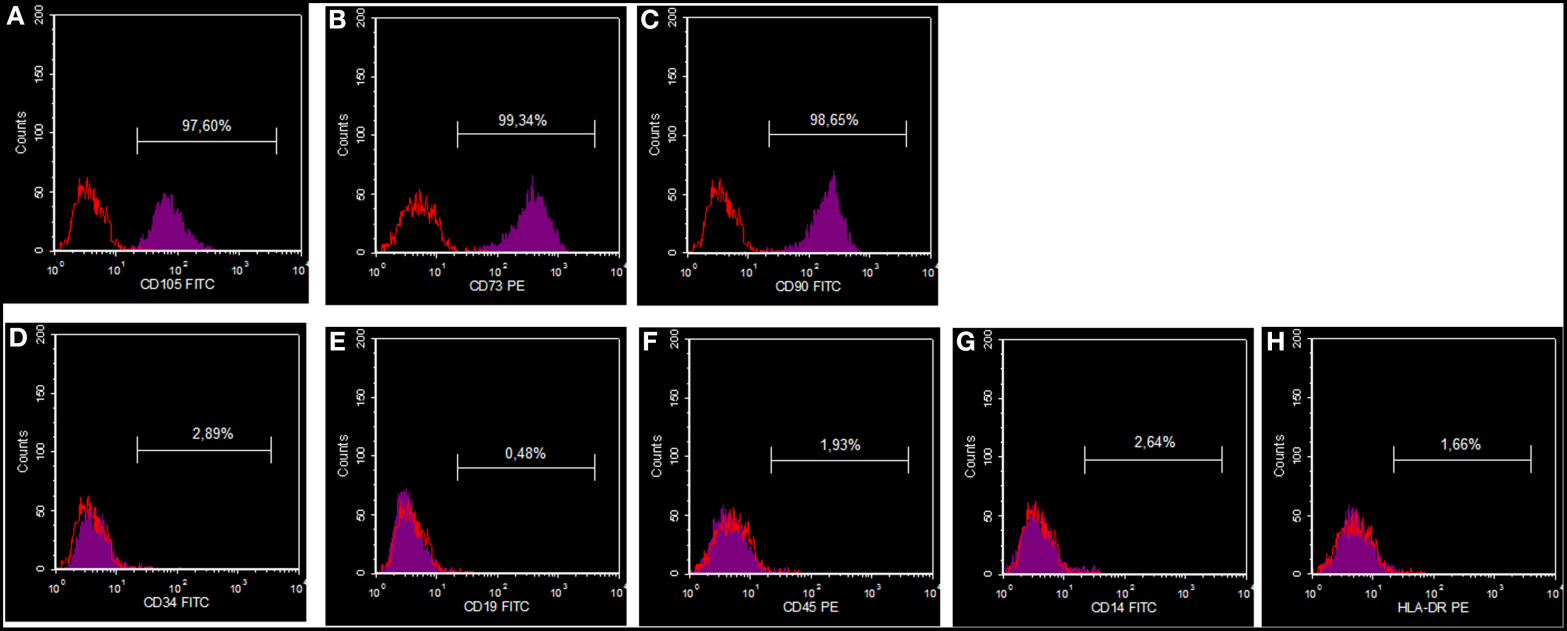
**DPSC phenotypic profile**. Cells showing a positive reaction for CD105 **(A)**, CD73 **(B)** and CD90 **(C)**, and a negative reaction for CD34 **(D)**, CD19 **(E)**, CD45 **(F)**, CD14 **(G)** and HLA-DR **(H)**.

The MSCs utilized for all additional experiments were characterized for CD105, CD14, CD19, CD34, CD45, CD73, CD90, and HLADR using flow cytometric analysis. It was found that these cells were highly positive for CD105, CD73, and CD90, and negative for CD34, CD19, CD45, CD14, and HLA-DR (Figure 1).

### MTT assay

**Figure 2:**
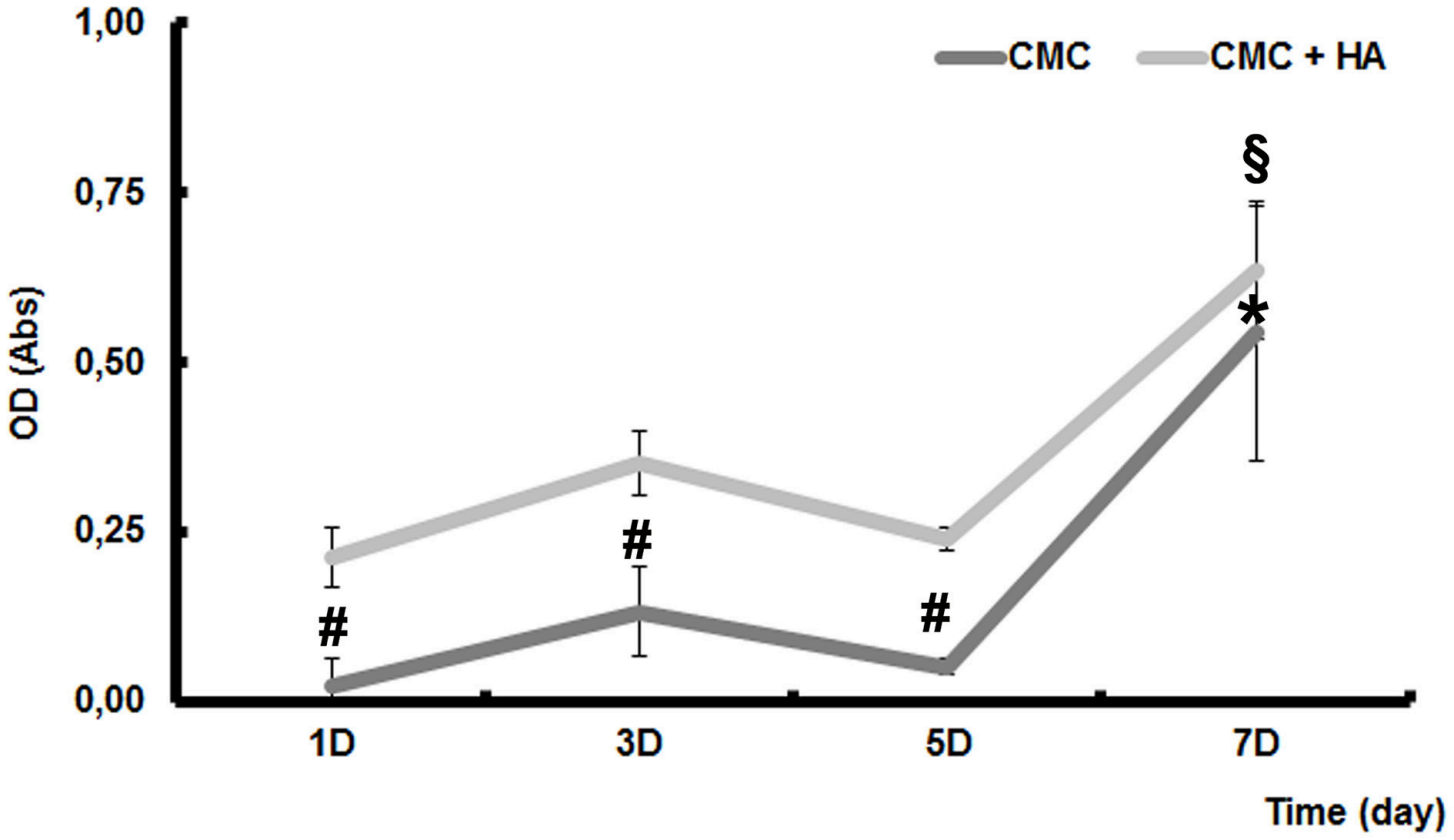
**MTT assay of DPSCs seeded on CMC and CMC-HA scaffold for 1, 3, 5, and 7 days**. Data were expressed as mean of percentage ± SD. ^*^represents a significant difference compared to DPSCs cultured for 1 day on CMC scaffold, P < 0.05. §represents a significant difference compared to DPSCs cultured for 1 day on CMC-HA scaffold, *P* < 0.05. ^#^ represents a significant difference of DPSCs cultured on CMC scaffold to DPSCs cultured on CMC-HA scaffold, *P* < 0.05.

In order to evaluate the biocompatibility of CMC and CMC-HA scaffolds cultured with DPSCs for 3, 5, and 7 days, an MTT assay was carried out. Cell viability results showed a high biocompatibility for CMC-HA material, while a lower cell viability is observed in CMC sample (Figure 2).

### High resolution scanning electron microscopy (HR-SEM)

To demonstrate the cell adhesion on both scaffold surfaces, high resolution scanning electron microscopic analysis was carried out.

**Figure 3:**
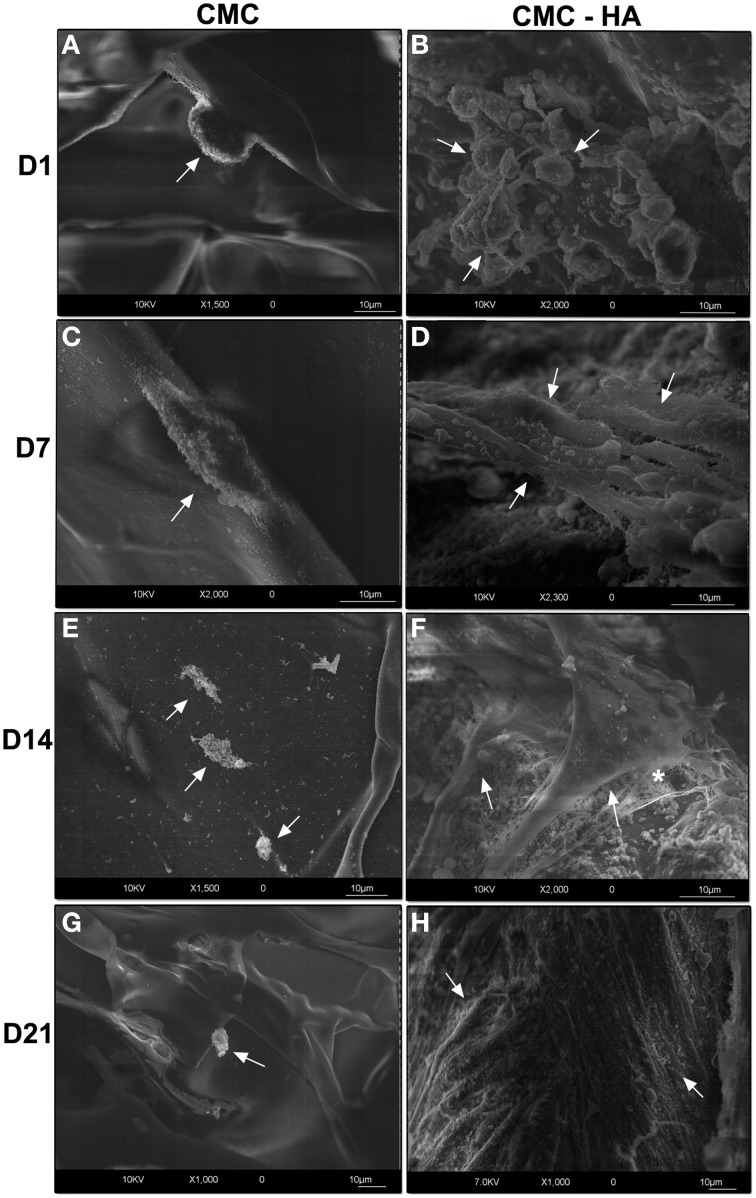
**HRSEM showing DPSCs on scaffold surface**. **(A)** Cells (arrow) on CMC scaffold surface after 1 day of culture (bar: 10 um); **(C)** DPSCs (arrow) on CMC surface after 7 days (bar: 10 um); **(E)** cell debris (arrows) at day 14 on CMC surface (bar: 10 um); **(G)** cell debris (arrow) after 21 days on CMC surface (bar: 10 um); **(B)** DPSCs (arrows) after 1 day of differentiation on CMC-HA material (bar: 10um); **(D)** CMC-HA scaffold showing DPSCs with a fibroblast shape (arrows) morphology after 7 days of culture (bar: 10 um); **(F)** DPSCs (arrows) on CMC—HA scaffold after 14 days of culture. Some extracellular matrix components (*) are detected (bar: 10 um); **(H)** CMC-HA hydrogel surface is completely covered by DPSCs (arrows) (bar: 10um).

CMC scaffold showed a smooth surface where just a few cells were weakly fixed on it after 1 day of culture (Figure 3A). They appeared with a round shape morphology (Figure 3A). After 7 days of differentiation, a few cells with fibroblast like morphology were detected (Figure 3C). Only cell debris were observed after 14 (Figure 3E) and 21 (Figure 3G) days of differentiation.

**Figure 4:**
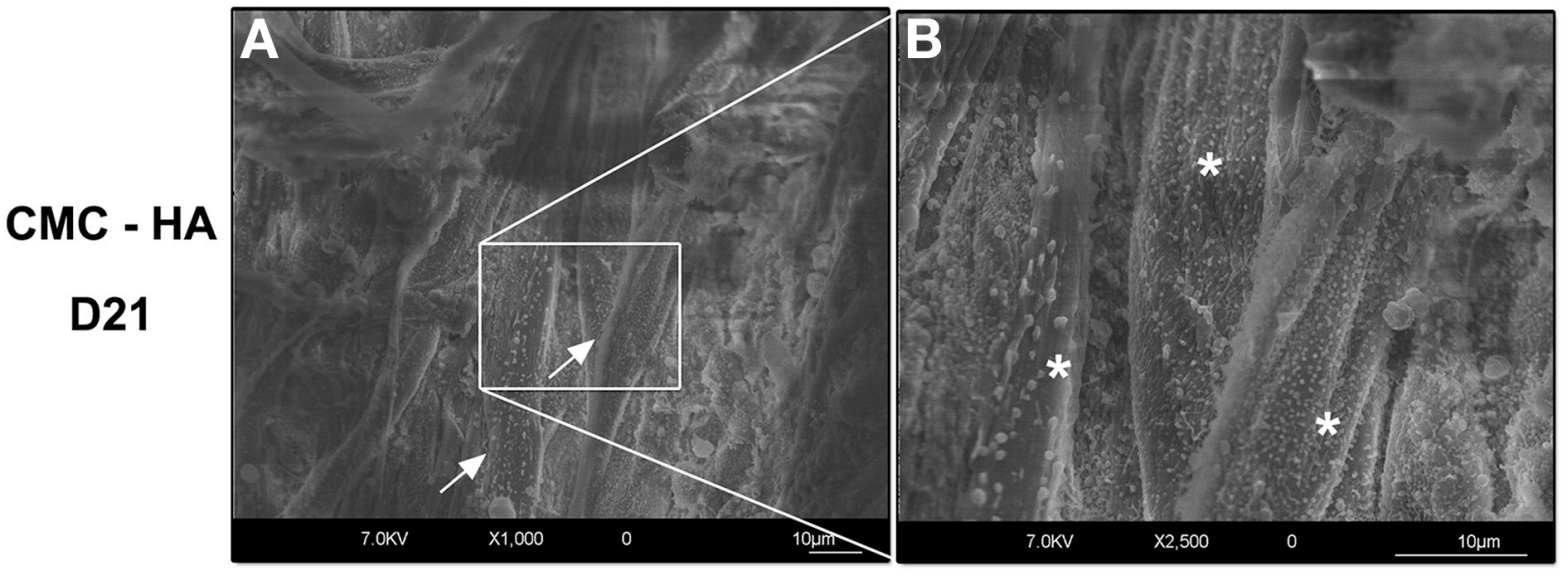
**(A)** DPSCs (arrows) completely covered the surface of CMC—HA hydrogel after 21 days of differentiation (bar: 10 um). **(B)** Higher magnification of the surface of cells characterized by several secretory vesicles (*) (bar:10um).

On the contrary, CMC-HA scaffold showed a rough surface due to HA crystals. After 1 day of incubation, several cells appeared with a round-shaped morphology (Figure 3B). After 7 and 14 days of differentiation, cells with a fibroblast like morphology were observed (Figures 3D,F). CMC-HA surface was completely covered by DPSCs at day 21 (Figures 3H, 4A). Several secretory vesicles were observed on the cell surface (Figure 4B).

### Light microscopy of DPSCs differentiated on CMC and CMC-HA hydrogels

**Figure 5:**
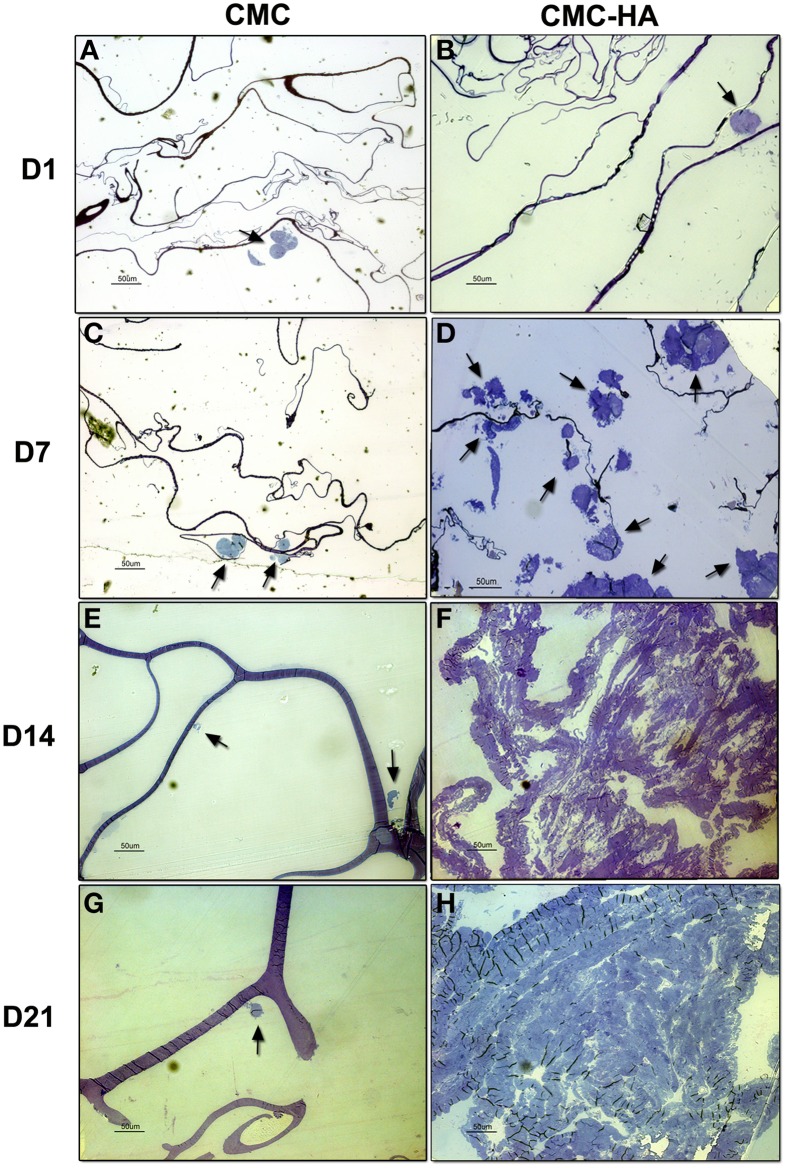
**Resin embedded sections of (A) DPSCs cultured on CMC hydrogel for 1 day**. Cells organized in small clusters (arrow) on scaffold surface (bar: 50 um); **(B)** DPSCs cultured on CMC-HA hydrogel for 1 day. Cells organized in small clusters (arrow) (bar: 50 um); **(C)** DPSCs cultured for 7 days on CMC hydrogel. Just a few cell (arrows) were detected (bar: 50 um); **(D)** DPSCs cultured on CMC-HA hydrogel for 7 days. Cells proliferated and spread out on scaffold surface (arrows) (bar: 50 um); **(E)** DPSCs grown on CMC scaffold for 14 days. Cellular debris were observed (arrows) (bar: 50 um); **(F)** After 14 days of culture DPSCs covered completely the surface of CMC-HA material (Bar: 50 um); **(G)** DPSCs grown on CMC hydrogel for 21 days. Cellular debris were detected (arrow) (bar: 50 um); **(H)** CMC—HA scaffold with DPSCs cultured for 21 days. Cells still covered surface of the material (bar: 50 um).

After 1 day of culture, sections of CMC samples showed small clusters of DPSCs on the surface of the material (Figure 5A). After 7, 14, and 21 days of culture, just a few cells were detected on the scaffold surface (Figures 5C,E,G).

Sections of CMC-HA hydrogels, showed small clusters of DPSCs on the material after 1 day of culture (Figure 5B). After 7 days of differentiation, several cells with a fibroblast-shaped morphology were detected on the scaffold (Figure 5D). By the end of Day 14 (Figure 5F) and 21 (Figure 5H), the hydrogel surface was completely covered by DPSCs.

Due to the low adhesion of cells on CMC based hydrogels, confirmed also by HRSEM analysis, we decided to perform the next experiments only on CMC-HA based hydrogels.

### TEM analysis of DPSCs differentiated on CMC-HA scaffold

To better visualize the ultrastructure of DPSCs cultured on CMC-HA hydrogel, a TEM analysis was carried out.

**Figure 6:**
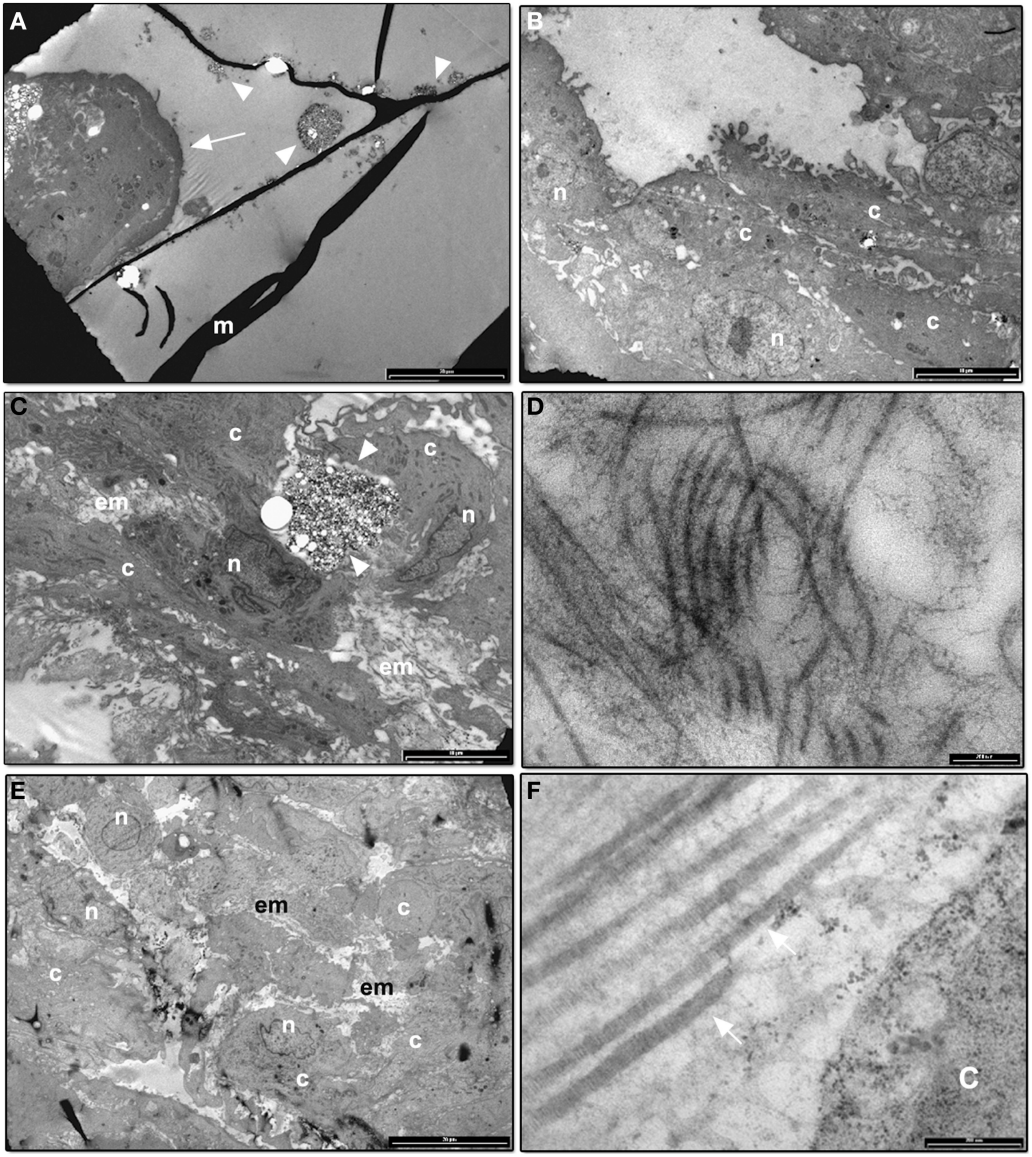
**(A)** TEM image of CMC-HA scaffold with DPSCs organized in cluster (arrow) after 1 day of culture (m: hydrogel material; arrowheads: HA crystals; bar: 20 um); **(B)** TEM image of DPSCs cultured on CMC-HA hydrogel for 7 days. Cells appear well-preserved (n, nucleus; c, cytoplasm; bar: 10 um); **(C)** TEM analysis of DPSCs cultured for 14 days. An initial deposition of the extracellular matrix (em) was observed (n, nucleus; c, cytoplasm; arrowhead, HA crystals; bar: 10 um); **(D)** High magnification of the fibrillary structures observed in the extracellular matrix of DPSCs cultured on CMC—HA for 14 days (bar: 200 nm); **(E)** TEM image of DPSCs grown for 21 days on CMC—HA hydrogel. Cells completely covered hydrogel surface (n, nucleus; c, cytoplasm; em, extracellular matrix; bar: 20 um). **(F)** High magnification of fibrillary structures observed in the extracellular matrix of cells culture on CMC—HA hydrogel for 21 days. A banding structure connected with type I collagen fibers is observed (arrow) (c, cytoplasm; bar: 200 nm).

Figure 6A shows DPSCs cultured for 1 day on CMC-HA scaffolds. Cells appeared organized in clusters, showing a well-preserved morphology (Figure 6A). CMC-HA hydrogel fibers with small crystals of HA were also detected (Figure 6A). By the end of the first week of differentiation, DPSCs were spread out on scaffold surface and they showed a fibroblast like morphology (Figure 6B). The nucleus and cytoplasmic organelles were well-preserved. No sign of extracellular matrix deposition was observed. After 14 days of differentiation, DPSCs showed several, fibrillary components in the extracellular space related to extracellular matrix (Figures 6C,D). By the end of the third week of differentiation, DPSCs completely covered the surface of the hydrogel (Figure 6E). In the extracellular matrix several fibrillary structure, with a banding organization, were observed (Figure 6F).

### Real time PCR analysis

To confirm osteogenic and odontogenic differentiation, the expression of some osteogenic markers such as ALP, RUNX2, COL1A1, SPARC, and odontogenic markers such as DMP1 and DSPP were analyzed in DPSCs cultured on CMC-HA samples for 1, 3, 5, 7, 14, and 21 days.

**Figure 7:**
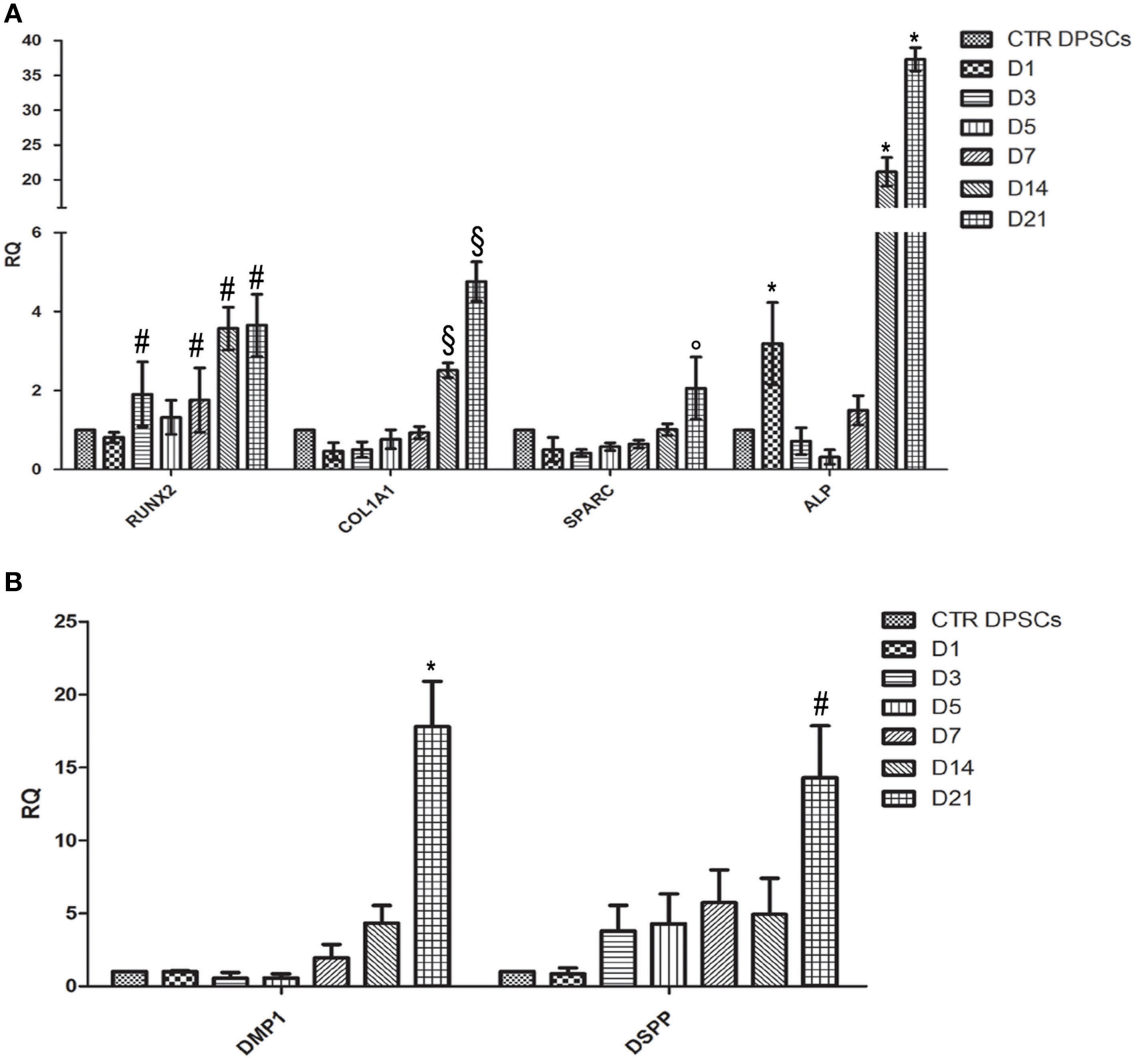
**(A)** Quantitative Real Time PCR analysis of mRNA of RUNX-2, COL1A1, SPARC, and ALP of DPSCs cultured on CMC-HA scaffold for 1, 3, 5, 7, 14, and 21 days. Each individual assay was performed in triplicates and expressed as mean ± SD; ^#^ represents a significant difference of RUNX-2 expression relative to control DPSCs (CTR DPSCs), *P* < 0.05; §represents a significant difference of COL1A1 expression relative to control DPSCs (CTR DPSCs), *P* < 0.05; ° represents a significant difference of SPARC expression relative to control DPSCs (CTR DPSCs), *P* < 0.05; ^*^represents a significant difference of ALP expression relative to control DPSCs (CTR DPSCs), *P* < 0.05; **(B)** Quantitative Real Time PCR analysis of mRNA of DMP-1and DSPP of DPSCs cultured on CMC-HA scaffold for 1, 3, 5, 7, 14, and 21 days. Each individual assay was performed in triplicates and expressed as mean ± SD; ^*^represents a significant difference of DMP1 expression relative to control DPSCs (CTR DPSCs), *P* < 0.05; ^#^ represents a significant difference of DSPP expression relative to control DPSCs (CTR DPSCs).

The transcription factor RUNX2 showed an increase of expression just after 7 days of culture, according to the first steps of osteogenesis (Figure 7A). A significant up-regulation of COL1A1 expression was detected at day 14 and 21 of differentiation, according to TEM images in which fibrillary structures resembling type I collagen protein were detected in the extracellular matrix. Transcript of ALP and SPARC were also significant higher after 14 and 21 days of differentiation (Figure 7A).

Regarding odontogenic differentiation, the expression of DMP-1 and DSPP showed a gradually up-regulation during the time of differentiation with the highest level at D21 compared to control samples (Figure 7B).

## Discussion

Highly proliferative and multi-lineage subpopulations of mesenchymal stem cells isolated from dental tissues are capable of differentiating toward odontoblast and osteoblast phenotypes (Gronthos et al., 2000; Miura et al., 2003; Tirino et al., 2011; La Noce et al., 2014; Pisciotta et al., 2015). These stem cells are readily available and easily accessible, making them promising tools for regenerative medicine.

Beside the well-known ability of DPSCs to be *in vitro* differentiated into functional osteoblasts producing extracellular and mineralized matrix in abundance (Riccio et al., 2010; Pisciotta et al., 2012), there are *in vivo* studies reporting the capacity of human DPSCs to reconstruct bone structures (d'Aquino et al., 2009b; Giuliani et al., 2013). These results suggest that DPSCs can be successfully utilized for dental pulp complex and periodontal tissue engineering.

In such strategies, the interaction between stem cells and the material applied as scaffold plays a critical role in the generation of a cell-friendly microenvironment, which must be conducive to the regeneration of dental structures (Conde et al., 2015).

The two categories of materials that are most commonly used in tissue engineering are synthetic polymers such as poly(lactic) acid (PLA) and poly(glycolic) acid (PGA) (Galler et al., 2011) and matrices derived from biological sources such as reconstituted collagen (Falconi et al., 2013; Focaroli et al., 2014; Ivanovski et al., 2014). In the last years, hydrogel systems were proposed as new biomaterials for minimally invasive surgery, repair of bone defects with irregular shape, such as periodontal defects, filling materials and *in vivo* delivery of bioactive signals or cells (Barbucci et al., 2010; Pasqui et al., 2012). Cellulose based hydrogels are well-known for their low toxicity, high biocompatibility, and low degradation rates. The main drawback is the lack of good mechanical proprieties. The presence of HA crystals in the scaffold offers suitable mechanical proprieties, and roughness which can increase cell adhesion (Geckil et al., 2010; Pasqui et al., 2012). The aim of this study was to evaluate the osteogenic and odontogenic differentiation of DPSCs cultured on a CMC-HA hydrogel and to verify the biological proprieties of this biomaterial which can subsequently be utilized in tissue regeneration. To our knowledge, the present experimental study is the first report demonstrating a favorable biological performance of CMC-HA hydrogels toward stem cell osteogenic and odontogenic differentiation.

Our cell viability assay of DPSCs seeded on CMC-HA showed a good biocompatibility of the material in agreement with previous studies on cellulose-based scaffolds (Pasqui et al., 2014). The MTT assay in particular demonstrated a slight increase in cell proliferation the first day of culture and a peak of proliferation at day 7, suggesting a period of adaption for the cells on scaffold surface prior to the proliferation phase. This behavior was already demonstrated for mesenchymal stem cells separated by bone marrow (Teti et al., 2012) and cultured on collagen-based scaffold (Focaroli et al., 2014). On the other end, cells seeded on CMC based hydrogel, although showing an increased metabolic activity up to day 7, had values of cell viability always lower than CMC-HA samples. Considering that the number of cells seeded on both materials was initially the same, we concluded that in CMC samples there was a weaker adhesion of cells to the scaffold surface and less proliferation during the time of incubation. These findings reflect one of the common problems associated with non-human polysaccharide based hydrogels (carboxymethylcellulose, chitosan, alginates, etc.) utilized for tissue engineering applications, characterized by a lack of bioactivity, which usually limits cell adhesion to biomaterials and further tissue integration (Shin et al., 2004; Jing et al., 2015). One approach to promote polysaccharide biomaterials interactions with surrounding tissue has been to tether cell-binding peptides to the biomaterial, through physical or chemical modification methods, to provide biological cues to mimic cell–extracellular matrix protein interactions (Jing et al., 2015).

Polysaccharide based hydrogels, designed for bone tissue engineering, need stronger mechanical proprieties to regenerate hard tissues (Pasqui et al., 2014; Mattei et al., 2015; Michel et al., 2015). To this aim, it has been demonstrated that the combination of hydroxyapatite with natural or synthetic hydrogels improves bioactivity, osteoinductivity, and osteoconductivity (Michel et al., 2015).

A previous investigation reports that the CMC-HA hybrid hydrogel showed the presence of HA crystal homogenously distributed inside and on the hydrogel surface, improving inherent mechanical and adhesive proprieties (Pasqui et al., 2012, 2014).

HR-SEM analysis confirmed a poor adhesion of DPSCs on the CMC surface. At the end of day 21, no cells were detected on the hydrogel surface. This result is apparently in contrast with cell viability data, where at day 7 a peak of cell viability was observed. We speculate that during the preparation of the sample for electron microscopy analysis, cells were lost due to a weak adhesion on the CMC scaffold.

HR-SEM images of the CMC-HA scaffold showed a good adhesion of cells after 1 day of culture. By the end of day 21, the cells covered the scaffold surface, showing a fibroblast-like morphology and several secretory vesicles on the cellular membrane, suggesting intense protein synthesis mainly connected with the differentiation process (Venugopal et al., 2010; Teti et al., 2012). TEM images demonstrated the presence of extracellular matrix fibrils resembling type I collagen protein at day 14 and 21, in agreement with the commitment of MSCs toward the osteogenic and odontogenic lineage (Riccio et al., 2010; Teti et al., 2012).

To fully confirm the osteogenic differentiation of DPSCs cultured on CMC-HA hydrogel, a Real Time PCR analysis was carried out to test mRNA expression of osteogenic markers such as ALP, RUNX2, COL1A1, and SPARC. RUNX2 is a transcription factor involved in osteoblastic differentiation and skeletal morphogenesis. It has been shown to affect the expression of type I collagen and SPARC by binding to the promoters of these genes. RUNX2 and COL1A1 are known to be early markers of osteoblastic differentiation while SPARC is involved in initiating mineralization and promoting mineral crystal formation during bone formation. ALP appears to be intimately related to pre-osseous cellular metabolism and to the elaboration of a calcifying bone matrix (Siffert, 1951).

The data obtained showed the expected expression profiles of the osteoblast phenotype. Results showed an up-regulation of all osteogenic markers after day 14 and 21, compared to control DPSCs. These data are in agreement with the temporal gene expression demonstrated during osteogenesis (Kulterer et al., 2007; Raggatt and Partridge, 2010; Fakhry et al., 2013). An up-regulation of RUNX2 was evident during the whole period of differentiation. COL1A1 reached a maximum of expression on day 21 in agreement with TEM images that showed the presence of extracellular fibrillary structures, resembling type I collagen protein. Also SPARC showed a nearly constant, up-regulated expression level with a peak on day 21 of differentiation.

To demonstrate odontogenic differentiation, the mRNA expression of DMP1 and DSPP genes was investigated by Real Time PCR. DMP1 plays crucial roles in the formation of dentin mineralized tissues (Ye et al., 2004). DSPP is a large protein which subsequently undergoes cleavage to generate two products: dentin sialoprotein (DSP) and dentin phosphoprotein (DPP) (Yamakoshi et al., 2006). DPP is thought to play a role during the nucleation of calcium phosphate while DSP has little or no effect on mineralization; its real function remains unclear (Yamakoshi et al., 2006). Our data demonstrated an upregulation of both odontogenic markers during the process of differentiation reaching peak levels at D14 and D21, corresponding to the phase of deposition of extracellular matrix and mineralization (Kulterer et al., 2007).

In conclusion, our *in vitro* data demonstrated that the CMC-HA hybrid hydrogel has suitable proprieties in supporting DPSCs adhesion, proliferation and osteogenic and odontogenic differentiation. This novel biomaterial could be a promising candidate for periodontal and dental pulp tissue engineering.

## Author contributions

Each author substantially contributed to experimental procedures. In particular, Dr. Teti and Prof. Orsini planned the research and experiments. Dr. Mazzotti, Dr. Dicarlo, and Dr. Durante were responsible for cell culture, differentiation treatment and hydrogel synthesis. Dott. Salvatore performed Real Time PCR data. Dott. Focaroli performed TEM analysis; Dr. Monica Mattioli Belmonte performed SEM observation. Prof. Orsini oversaw the whole research. All authors equally and competently contributed to the draft.

## Funding

This work was supported by Fondazione del Monte di Bologna e Ravenna.

### Conflict of interest statement

The authors declare that the research was conducted in the absence of any commercial or financial relationships that could be construed as a potential conflict of interest.
